# Stop in Time: How to Reduce Unnecessary Antibiotics in Newborns with Late-Onset Sepsis in Neonatal Intensive Care

**DOI:** 10.3390/tropicalmed9030063

**Published:** 2024-03-19

**Authors:** Domenico Umberto De Rose, Maria Paola Ronchetti, Alessandra Santisi, Paola Bernaschi, Ludovica Martini, Ottavia Porzio, Andrea Dotta, Cinzia Auriti

**Affiliations:** 1 Neonatal Intensive Care Unit, “Bambino Gesù” Children’s Hospital IRCCS, 00165 Rome, Italy; mariapaola.ronchetti@opbg.net (M.P.R.); alessandra.santisi@opbg.net (A.S.); ludovica.martini@opbg.net (L.M.); andrea.dotta@opbg.net (A.D.); 2 PhD Course in Microbiology, Immunology, Infectious Diseases and Transplants (MIMIT), Faculty of Medicine and Surgery, “Tor Vergata” University of Rome, 00133 Rome, Italy; 3 Microbiology Unit, “Bambino Gesù” Children’s Hospital IRCCS, 00165 Rome, Italy; paola.bernaschi@opbg.net; 4 Clinical Laboratory Unit, “Bambino Gesù” Children’s Hospital IRCCS, 00165 Rome, Italy; ottavia.porzio@opbg.net; 5 Department of Experimental Medicine, University of Rome “Tor Vergata”, 00133 Rome, Italy; 6 Casa di Cura Villa Margherita, 00161 Rome, Italy; cinzia.auriti@gmail.com; 7 Faculty of Medicine and Surgery, Saint Camillus International University of Health Sciences, 00131 Rome, Italy

**Keywords:** infection, biomarkers, sepsis definition, clinical score, blood culture, time to positivity, automatic stop order, LOS, neonates

## Abstract

The fear of missing sepsis episodes in neonates frequently leads to indiscriminate use of antibiotics, and prescription program optimization is suggested for reducing this inappropriate usage. While different authors have studied how to reduce antibiotic overprescription in the case of early onset sepsis episodes, with different approaches being available, less is known about late-onset sepsis episodes. Biomarkers (such as C-reactive protein, procalcitonin, interleukin-6 and 8, and presepsin) can play a crucial role in the prompt diagnosis of late-onset sepsis, but their role in antimicrobial stewardship should be further studied, given that different factors can influence their levels and newborns can be subjected to prolonged therapy if their levels are expected to return to zero. To date, procalcitonin has the best evidence of performance in this sense, as extrapolated from research on early onset cases, but more studies and protocols for biomarker-guided antibiotic stewardship are needed. Blood cultures (BCs) are considered the gold standard for the diagnosis of sepsis: positive BC rates in neonatal sepsis workups have been reported as low, implying that the majority of treated neonates may receive unneeded drugs. New identification methods can increase the accuracy of BCs and guide antibiotic de-escalation. To date, after 36–48 h, if BCs are negative and the baby is clinically stable, antibiotics should be stopped. In this narrative review, we provide a summary of current knowledge on the optimum approach to reduce antibiotic pressure in late-onset sepsis in neonates.

## 1. Introduction

Antibiotic exposure during the first days of life has been associated with the emergence of multidrug-resistant (MDR) bacteria, the onset of several difficult to treat infections, disruption of developing individual microbiota, and the greater ease of developing chronic illnesses later in life. Therefore, the consequences of improper use of antibiotics in neonatal and early childhood are increased mortality, increased acute and chronic morbidity, and a marked increase in healthcare costs [1]. Sepsis rule-out is the primary cause of antibiotic overuse in neonates and infants in Neonatal Intensive Care Unit (NICU) settings [2].

The first development point in neonatal sepsis comes between early onset sepsis (EOS), which usually arises before 72 h of life, and late-onset sepsis (LOS), which arises after 72 h of life. The distinction between the two types of infection is therefore based on the different natures of the responsible bacteria, the mode of transmission from mother to newborn, and the timing of the onset of symptoms, with this classification influencing management and antibiotic therapy. In EOS, there is a transplacental passage of bacteria from the mother to the fetus (the vertical transmission of the infection, as with Listeria infections) or an ascending infection via the maternal genital tract (as in *Group B Streptococcus*—GBS—and *Escherichia coli* infections). LOS infections are mainly caused by organisms horizontally acquired from the environment, either from the community or from the hospital environment (in the form of Staphylococcal infections acquired from surfaces, health-care workers’ hands, central venous catheters, or other devices), and also include cases of neonates presenting to the emergency department with suspected sepsis [3]. In recent literature, the EOS-risk calculator has been the most discussed approach to reducing the start of antibiotic therapy for suspected EOS in late preterm and term infants [4]. Conversely, a similar software is unavailable for LOS episodes in regard to reducing unnecessary antibiotics. Therefore, even experienced neonatologists often start antibiotics because of the fear of missing a true sepsis case without clear diagnostic criteria. Therefore, we must focus on stopping antibiotics as soon as possible in LOS episodes when they are no longer required.

The aim of this narrative review is to focus on interventions to improve the decision-making process regarding the stoppage of antibiotics in late-onset sepsis and to summarize the available evidence.

## 2. Materials and Methods

This narrative review was produced by searching for articles in the PubMed database and matching the terms “sepsis” and “neonate” with “late-onset”, “antibiotics”, or “antibiotic stewardship”. All retrieved articles written in English and published before 30th December 2023 were analyzed without imposing restrictions on date or year, location, study design, study aims, or inclusion/exclusion criteria. We also screened reference lists of identified studies, and additional references for this review were identified by each author based on their knowledge of the field.

## 3. From the Definition of Sepsis to the Use of Clinical Scores

In contrast to adult and pediatric sepsis, which employ organ failure, neonatal sepsis has a wide range of diagnoses based on microbiological culture, laboratory testing, and clinical symptoms [5]. “Culture-proven sepsis” or “definite sepsis” should be distinguished from “probable sepsis” or “clinical sepsis”, followed by “possible sepsis”, as defined by some studies [5]. In particular, the diagnosis of neonatal sepsis is heavily related to the isolation of pathogens from blood and the prescription of antibiotic therapy, with a lack of a consensus about the neonatal definition [6]. Consequently, especially without evidence of a positive blood culture, the most frequently used criterion is that of a combination of clinical and laboratory symptoms, with there being an enormous variability of behaviors and prescriptive policies between different NICUs worldwide [7]. Standardizing the criteria for the evaluation of a possible LOS and the need to administrate antibiotics is a crucial target and can help to reduce unnecessary doses [8]. While the phrases “possible sepsis” or “suspected sepsis” may be suitable during the first examination and administration of empirical antibiotics, they cannot be provided as a definitive diagnosis of certainty and prolonged treatment. The diagnosis should always be reconsidered in these cases, as antibiotic treatment may not be warranted.

In 2015, the Third International Consensus Definitions for Sepsis and Septic Shock (Sepsis-3) defined sepsis as a “*life-threatening organ dysfunction caused by a dysregulated host response to infection*” that is associated with a Systemic Inflammatory Response Syndrome (SIRS) and can evolve towards multiple organ failure [9]. The task force focused on adult patients, with the aim of developing similar updated definitions for pediatric populations in a second moment. Previously, Hofer and colleagues evaluated whether the SIRS and sepsis criteria were applicable to infected full-term newborns, finding that only 53% of the septic patients with positive cultures fell into the shared definition [10].

Indeed, the definition of SIRS includes many parameters inherent to the physiology of the newborn at birth (tachycardia, tachypnea, increase in bilirubin above 5 mg/dL, and oliguria). The evolutionary characteristics of the SIRS were described using a score called the Sequential [Sepsis-related] Organ Failure Assessment score (SOFA score), which is unsuitable for newborns for the above-mentioned reasons. Furthermore, newborns were considered to be among the population under the age of 18, preterm newborns were excluded, and neonatologists with experience with newborns were not included among the convened experts [9].

The current definition of sepsis focuses on the presence of organ dysfunction (host response) as a major source of morbidity and mortality. Newborns requiring intensive care at birth challenge the ability to make a diagnosis of organ dysfunction, as there is no baseline data available from which to measure postpartum change. Moreover, preterm infants may be unstable from birth regardless of the presence of infections. It is not clear what the most accurate method to screen especially preterm newborns for organ dysfunction might be. Positive blood cultures are considered the gold standard for diagnosis, and the rate of positive blood cultures in neonatal sepsis work-up has been reported to be around 9%, meaning that, in most cases, sepsis is ruled out based on (truly) negative blood cultures [11].

Wynn and Polin suggested that the work undertaken to develop the Sepsis-3 screening in 2016 could be adapted to the newborn. They proposed an objective, electronic health record-automated, neonatal sequential organ failure assessment (nSOFA) score to predict mortality from LOS in preterm, very low birth weight (VLBW) infants [12]. The nSOFA employs three categorical scores (total score range 0–15) to objectively describe dynamic changes in (1) the need for mechanical ventilation and oxygen (score range 0–8); (2) the need for inotropic support (for presumed adrenal insufficiency or catecholamine-resistant shock) (score range 0–4); and (3) the presence and degree of thrombocytopenia (score range 0–3) (Table 1). A score higher than four in LOS was associated with higher mortality [12], as confirmed by Fleiss et al. [13] and Poggi et al. [14]. In particular, the likelihood ratio for mortality progressively increased as the nSOFA score increased (2-fold with a nSOFA score ≥2, 4-fold with a score ≥6, 8-fold with a score ≥8, and 16-fold with a score ≥10) [13]. An abrupt change in the nSOFA score could be the turning point in stopping antibiotics in the absence of a positive culture result despite the lack of evidence about LOS to date.

However, the nSOFA score will likely expand to include other measures of organ dysfunction that are shown to be significant for sepsis mortality in evidence-based studies. For example, the central nervous system, liver, and renal dysfunction are included in the adult SOFA score. Currently, measurements of these systems in newborns are more problematic. In adults, total bilirubin of 5 mg/dL represents liver dysfunction; however, this amount would remain absolutely within normal limits for newborns for several days after birth. It is certainly more accurate in late-onset sepsis than in early onset samples because, in preterm infants, many symptoms of respiratory or cardiovascular instability at birth are present regardless of infections. A change in nSOFA from a baseline nSOFA could be more accurate for describing a worsening trend. This is in part due to components such as platelet count, which can be lower in the first days of life in, for example, those infants born to mothers with preeclampsia. Additionally, postnatal steroids can influence the score, and there are different reasons for the need for postnatal steroids in neonates, such as in the case of intubated infants with severe chronic lung disease or those with stridor.

Conversely, Sokou et al. proposed the new Neonatal Sepsis Diagnostic (NeoSeD) score, which includes gestational age, CRP, considerable skin discoloration, liver enlargement, neutrophil left shift, and a thromboelastometry extrinsically activated (EXTEM) assay. The authors reported an excellent discrimination capacity for sepsis and septic shock, which was significantly higher compared to nSOFA scores [15]. This item should be externally validated, but we think the need for a thromboelastometry assay can limit the widespread use of this tool.

Very recently, a global neonatal sepsis observational cohort study (NeoOBS), including data from low-and middle-income countries, highlighted the urgent need for clinical trials to inform antibiotic use for neonatal sepsis globally. A total of 3204 infants were enrolled, with a median birth weight of 2500 g (IQR 1400 to 3000) [16]. The authors developed (1) a baseline NeoSep Severity Score to predict 28-day mortality from factors known at sepsis presentation (Table 2) and (2) a NeoSep Recovery Score to predict the daily risk of death while treated with intravenous antibiotics from daily updated assessments of clinical status (Table 3). In particular, a NeoSep Recovery Score ≥4 was the most discriminative of mortality [16] and should be investigated for use in antibiotic de-escalation.

These clinical scoring tools have been demonstrated to correlate well with mortality, but prospective studies validating them in antibiotic stewardship are lacking. Indeed, the prompt improvement of these clinical scores can objectively help the clinician decide to stop antibiotics safely while awaiting a negative blood culture result.

## 4. Does the Magic Biomarker Exist?

The impact of sepsis on pediatric mortality makes interventions to contain its onset and evolution critical; however, early, targeted therapy has emerged as the cornerstone of severe sepsis treatment bundles in adults, children, and especially in neonates. A prospective study was conducted at the Neonatal Department of the Children’s Hospital of Philadelphia from September 2014 to February 2018 to evaluate the relationship between the timing of initiation of antibiotic therapy at the suspicion or diagnosis of sepsis and mortality. Among the 1946 examples of sepsis work-up, 128 episodes of definite sepsis were identified in 113 neonates. Prolonging the interval between suspected or definite diagnosis and the first dose of antibiotic administration was associated with a significantly increased risk of death at 14 (OR, 1.47; 95% CI, 1.15–1.87) and 30 days (OR, 1.47; 95% CI, 1.11–1.94), as well as fewer inotrope-free days (incidence rate ratio, 0.91; 95% CI, 0.84–0.98). There was no association between days without mechanical ventilation or length of hospital stay. Therefore, the authors concluded that, among infants with sepsis, a delay in the initiation of antibiotic therapy is an independent risk factor for death and cardiovascular dysfunction [17].

These data lead doctors to frequently administer antibiotics because, in the neonate, the uncertainty that accompanies the diagnosis of sepsis is high, and this translates into a very low treatment threshold. The lack of defined guidelines in critical clinical situations has also generated an intrinsic tendency towards overtreatments in NICUs and the difficulty in suspending antibiotics administration once it has started in all age categories of patients, especially in neonates. In data from the multicenter NO-MAS-R study cited above, 80% of the total 405 newborns receiving antibiotic treatment on the day of observation had received prolonged therapy for more than 72 h, regardless of the results of the cultures, with a median duration of 7 days of therapy [2].

Therefore, a major issue would seem to be determining when to start. As the stakes are high, delay is equal to increased mortality; if suspicion is high, it is necessary to start immediately. Additionally, we must also ascertain when stopping in necessary, and whether biomarkers can be trusted after 48h. The difficulty in answering these questions leads to overtreatment or excessively prolonged treatment in a large proportion of uninfected patients.

Obtaining a “magic biomarker” with high sensitivity and specificity and high negative predictive values might aid in preventing the catastrophic effects of prolonged antibiotic treatments in neonates. Furthermore, a small blood volume would increase the possibility of using this ideal biomarker even in those with low birth weights, such as a rapid laboratory turnaround time, 24 h bedside access, and a fair price. To date, no single biomarker has been found that meets most of these requirements, and the magic biomarker seems not to exist or has not yet been discovered [18].

Although C-reactive protein (CRP) and procalcitonin (PCT) are the most widely used biomarkers of neonatal sepsis, their accuracy is still controversial [19]. Further biomarkers, such as interleukin 6 (IL-6), interferon-gamma inducible protein 10 (IP-10), interleukin 10 (IL-10), neutrophil gelatinase-associated lipocalin (NGAL), pentraxin 3 (PTX3), presepsin (P-SEP), and lipopolysaccharide-binding protein (LBP), have been measured for the diagnosis of late-onset sepsis, but their clinical routine use in response to the antibiotic therapy is still an object of study [20]. In recent years, the use of P-SEP has spread to many NICUs because of the possibility of measuring it with a point-of-care device and the apparent absence of confounding factors that influence its levels [21,22]. Point-of-care methods are also available for CRP and PCT. Therefore, it is often the choice of the individual NICU regarding which biomarker to use based on “experience practice” and economic reasons due to kit costs: PCT is substantially more expensive than CRP, and cost-effectiveness studies about the three markers (CRP, PCT, and P-SEP) are not available in this population.

The dilemma is whether inflammatory indicators are game changers or merely gimmicks in LOS, as in the case of early onset sepsis [23].

### 4.1. C-Reactive Protein

CRP is an acute-phase reactant synthesized by the liver in response to inflammatory cytokines (primarily interleukin 6) generated by white blood cells reacting to microbial pyrogens, with a cut-off between 0.5 and 1 mg/dL in most studies about late-onset infections [24].

CRP rises 10–12 h after pathogen exposure and peaks at 48–72 h [25]. CRP levels return to normal levels after 3–7 days, but levels can be affected by other non-infectious inflammatory stimuli (such as perinatal asphyxia or major surgery) [26].

When commencing antibiotic treatment in neonates with suspected LOS, NICE guidelines recommend always monitoring baseline CRP levels [27]. Sequential CRP measurement may help in the early diagnosis of microbial infections and evaluate the response to therapy, allowing for the discontinuation of antibiotic therapy when CRP drops [28]. Two CRP levels < 1 mg/dL obtained 24 h after the onset of symptoms indicate that a bacterial infection is unlikely, justifying the suspension of antibiotic therapy [29,30].

Current evidence suggests that using CRP alone, or combined with other biomarkers, can allow neonatologists to confidently discontinue antibiotics early (24–48 h after onset) in the suspected infection without waiting for definitive microbiological results as long as the infant remains clinically well, although this has not been demonstrated by a specific randomized controlled trial. However, there is currently insufficient evidence to monitor CRP to stop the antibiotic therapy in late-onset sepsis.

The still active “Using Biomarkers to Optimize Antibiotic Strategies in Sepsis” study (Clinical Trials ID number: NCT02207114) is a US-based randomized controlled trial proposing to evaluate the potential impact of a biomarker-based algorithm on reducing unnecessary antibiotic use in different adult and pediatric/neonatal intensive care units. The authors are dosing nine blood biomarkers (including CRP and PCT) in two groups: the experimental group, where the biomarker-based algorithm, along with the patient’s biomarker assay results, are given to the clinical team to assist them in deciding to continue antibiotics or not, and the control group, where biomarkers’ results are not shared with the subject’s medical team. To date, details of enrolled neonates are not available, and the study has not resulted in any recruitments [31].

### 4.2. Procalcitonin

PCT is the precursor of the hormone calcitonin: the PCT gene (CALC-1) is almost exclusively expressed by neuroendocrine thyroid C cells in normal circumstances, while generated PCT is stored in the Golgi apparatus, explaining the relatively low amounts detected in the bloodstream. CALC-1 is upregulated and consequently expressed in all cells of the organism during systemic infections, resulting in the release of increased levels of PCT into the circulation [32].

PCT was sensitive enough to detect sepsis episodes much earlier than CRP [28] because it is detecTable 3 h after exposure, peaking by the 6 h mark [25]. After about 12 h, a plateau is attained, and PCT levels return to normal after 2–3 h. The interpretation of procalcitonin values in neonates is complicated by a physiological increase in the first 48 h of life and other perinatal factors (such as chorioamnionitis, hypoxia, perinatal asphyxia, and maternal pre-eclampsia) that can also lead to serum PCT values comparable to septic neonates. Afterward, a normal PCT (cut-off: 0.5 µg/L using the BRAHMS PCTTM assay) has a good negative predictive value for sepsis [26].

The Neonatal Procalcitonin Intervention Study (NeoPInS) found that a procalcitonin-guided decision-making approach dramatically decreased antibiotic treatment time for early onset study. In this multicentre randomized controlled trial, 1710 neonates born after 34 weeks of gestational age, and who had suspected early onset sepsis in the first 72 h of life, were enrolled and randomized to receive procalcitonin-guided treatment or standard care by the treating physician. For the PCT group, the duration of antibiotic therapy was reduced. No sepsis-related deaths occurred, and 9 (<1%) of 1710 neonates had a possible re-infection [26]. To date, there is still no clear evidence that a similar PCT-guided approach in LOS could reduce antibiotic overtreatment in neonates.

The current PROABIS Study (Procalcitonin and Duration of Anti-Biotic therapy In Late Onset Sepsis of Neonate) is a multicenter randomized controlled open trial comparing the efficacy of a PCT guided strategy (superiority aspect) and safety (non-inferiority aspect) versus usual strategy in LOS of the neonate, still recruiting neonates in France at time of writing (Clinical Trials ID: NCT03730636). The included infants are neonates born after 24 weeks of gestation, aged over 96 h of life, with a weight at the inclusion of more than 700 g. After inclusion, patients are randomly assigned (in a 1:1 ratio) to the duration of antibiotic therapy, according to PCT guidance (experimental group), or to the standard of care (control group). PCT concentration is measured every two days, and antibiotic therapy is stopped when the PCT level reaches a value equal to or below 0.5 ng/mL. The primary outcome is to evaluate the efficacy of the PCT-guided antibiotic strategy, with the number of days between the start and end of treatment, including treatment of the recurrence (if any). Secondary outcomes will include the mortality rate at 28 days following randomization and the days alive without any antibiotics at Day 28 [33].

### 4.3. Interleukin-6 and Interleukin-8

Interleukin-6 (IL-6) and interleukin-8 (IL-8) are multifunctional cytokines that have a role in immune response, hemopoiesis, and acute-phase responses.

IL-6 is one of the most studied cytokines in sepsis; its circulating levels rise rapidly in response to infection within 2 h after the onset of bacteremia, with a peak at approximately 6 h, and finally declining over the following 24 h. Divergent results have been published for neonates with early onset sepsis, ranging from decreased IL-6 production in term neonates, which is even more prominent in preterm infants, to IL-6 values similar to those observed in adults [34]. Küng et al. recently analyzed 8.488 IL-6 values in 1.695 neonates, including 752 very-preterm infants and 701 very-low-birthweight infants. They reported that serum IL-6 could be used with high accuracy to detect sepsis in neonates with the cut-off values of 80 pg/mL on the first day of life, 40 pg/mL in the first week (days 2–7), and 30 pg/mL after seven days of life [35].

In late-onset sepsis, Ng et al. serum IL-6 concentrations quickly fall to undetectable values during antibiotic treatment, and the high sensitivity (98%) and negative predictive values (98%) of IL-6 and CRP (together considered) indicated that antibiotics could be confidently discontinued at 48 h without waiting for microbiological results, providing that the infants are in good clinical condition [36].

Similarly, interleukin-8 (IL-8) is involved in the activation and chemotaxis of neutrophils, and it has been reported to guide diagnoses and stratify the severity of neonatal infections [37]. Serum IL-8 has moderate accuracy for diagnosing neonatal sepsis, according to the results of a meta-analysis including seven studies (taking both EOS and LOS), with a peak within 1–3 h of infection and a half-life of less than 4 h [38]. A threshold value of 70 pg/mL for IL-8 was determined in prior research on EOS using a receiver operating characteristic curve analysis of 882 IL-8 values in full-term and preterm infants [39]. However, the used cut-off values of IL-8 widely ranged from 0.65 to 300 pg/mL, and the differences in the cut-off might be due to the measurement method and the onset time of neonatal sepsis [38].

To date, there are no specific studies regarding the role of IL-6 and IL-8 in stopping empirical treatment, specifically in LOS episodes, identifying a specific cut-off or decrease to consider. As a result, further studies are needed to determine if an interleukin-guided step can safely reduce antibiotic overuse.

### 4.4. Presepsin

Presepsin is the soluble fragment of the CD14 receptor expressed on the cell wall of monocytes and macrophages. It functions as a receptor for the complex lipopolysaccharides-lipopolysaccharide-binding proteins (LPSs-LBPs) on the outer wall of Gram-negative bacteria. After coming in contact with bacteria, CD14 triggers an intracellular signal cascade mediated by Toll-like receptor 4 (TLR4), resulting in an inflammatory response against the microbe. P-SEP is formed when plasma proteases cleave the soluble fragment of CD14 (sCD14) during this specific inflammatory reaction, being released into the serum and measured [21,40]. Therefore, P-SEP production is linked to innate immunity and phagocytosis, induces the release of cytokines in cases of sepsis, increases significantly, activates the inflammatory cascade in patients with sepsis, and seems to be the earliest marker of sepsis [21].

Indeed, P-SEP is present in low concentrations in serum in healthy persons but rises in response to bacterial infections in 2 h, peaks in 3 h, and has an 8 h halftime faster than CPR and PCT, also in relation to the severity of the infection. Since serum P-SEP concentrations fall progressively throughout antibiotic administration, this biomarker may also play a role in assessing the response to the therapy [41].

Recent studies suggest that P-SEP may be used as a more accurate biomarker in neonatal sepsis than CRP and PCT [42]. According to the results obtained by Pietrasanta et al., in neonates of any gestational age (GA), during the first episode of suspected sepsis, either EOS or LOS P-SEP levels were well correlated with the severity of disease, being higher in neonates with septic shock (median 1557.5 pg/mL) and sepsis (median 1361 pg/mL) compared to those with infection in other body sites (median 977.5 pg/mL) at the moment when the infection was firstly suspected (*p* < 0.01) [43].

Different authors have indicated that serum P-SEP levels decrease gradually throughout the antibiotic treatment of both EOS and LOS [44,45,46]. However, cut-off values, specificity, and sensitivity varied substantially between investigations in relation to the infection type (often both EOS and LOS taken together) and methods of analysis (type of samples, plasma or whole blood) [21]. To date, there are no targeted studies on the role of P-SEP in weaning patients from empirical antibiotics, specifically in LOS episodes. Therefore, further research should be performed to evaluate whether presepsin can support medical decisions on the start and end of antibiotic therapies in the newborn during sepsis.

## 5. Blood Cultures Are the Diagnostic Gold Standard

**Figure 1:**
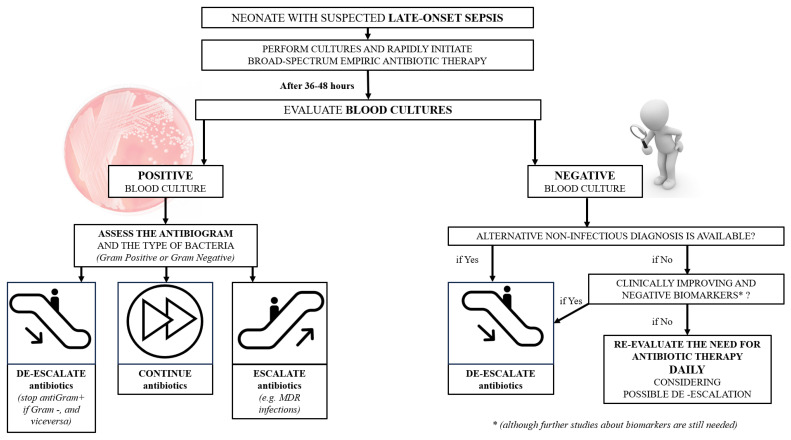
Flow-chart of management of neonates with late-onset sepsis.

Blood cultures (BC) are considered the gold standard for diagnosing bloodstream infections and are also a cornerstone in suspending antibiotic therapy (Figure 1). However, their performance depends on (1) pre-analytical factors (i.e., number of blood cultures, volume per blood culture bottle, and sites of blood collection), (2) analytical factors (i.e., accurate detection of pathogens), and (3) post-analytical factors (i.e., timely communication of results to clinicians providers and communication of possible contaminants) [47].

Currently, only 9% of blood cultures carried out to exclude sepsis are positive, indicating that there should be clinical symptoms that must guide the performance of the blood culture [11].

Most studies suggest that a blood volume of 1 mL is sufficient for neonates. However, sometimes, a volume of 0.5 mL is suggested, as this is the minimal volume validated for BC diagnostics, even if inadequate blood volume increases the probability of false-negative culture findings. This is linked to a higher proportion of contaminants [48]. In late-onset sepsis (LOS) only one aerobic BC bottle is usually processed in most NICUs, whereas few sites routinely add anaerobic BC bottles [49].

Furthermore, it is sometimes very difficult to obtain a blood volume sufficient for more than one BC bottle in neonates and critically ill infants. Therefore, smaller BC bottles were introduced to provide a favorable broth-to-blood volume ratio to promote the growth of most pathogens [48].

In the presence of a central venous catheter, a dual catheter and peripheral blood cultures should always be utilized, if possible. Most of the episodes demonstrated growth from both sites, and the risk of contamination should be considered when pathogens grow from a sole site [50].

### 5.1. Time to Positivity

Traditionally, empiric antibiotic duration ranges from 48 to 72 h [51], but developing more sensitive automated BC detection systems has shortened the time of positivity (TTP) for most cases [47].

Mukhopadhyay et al. reported in the US a median TTP of 23.5 h (IQR 18.4–29.9), with most LOS cases (364/428, 85%) being positive within 36 h. With the exception of coagulase-negative staphylococci (CoNS), 275/294 (93.5%), BCs were positive by 36 h. In a multivariable model, CoNS isolation and previous antibiotic treatment were significantly associated with increased odds of TTP > 36 h [52]. After 36 h, the probability of detecting a bacterial pathogen was 1.8% (95% CI: 1.4–2.2); in the case of a non-CoNS pathogen, it was 0.5% (95% CI: 0.3–0.7) [52]. Similarly, Guerti et al. (Belgium) found a median TTP of 21.2 h (IQR 13.4–31.6) in episodes of suspected LOS [53], while Abdelhamid reported a median TTP of 24 h in suspected LOS episodes in Egypt [54].

TTP varies by pathogen, with Dierig et al. reporting that median TTP values were shortest for *E. coli* and GBS (approximately 9 h each) and longest for *S. aureus* and CoNS (14 and 16 h, respectively) [55].

### 5.2. Repeat Blood Cultures

Repeat blood cultures should be obtained to demonstrate pathogen eradication or to distinguish contamination from true bacteria. In particular, they can have a high diagnostic value to document the clearance of *S. aureus* bacteremia [47]. The failure to sterilize the BC shows that the antibiotics used are ineffective against the infecting organism or that an unidentified infectious focus exists.

The existence of negative cultures upon repeat collection, serial trends of biomarkers, and the neonate’s overall look can all be used to dictate therapy duration in difficult-to-treat cases.

### 5.3. Narrowing the Spectrum with PCR Assays

Blood cultures can have a crucial role also in switching multiple antibiotic therapies towards monotherapy, narrowing the spectrum of our treatment when cultures and susceptibility tests’ results become available [56].

Rapid identification of microorganisms from blood cultures may lead to earlier intervention with appropriate antimicrobial and antifungal therapy. Real-time PCR has the potential to be a valuable additional tool for the diagnosis of neonatal sepsis, as confirmed in EOS [57].

The FilmArray blood culture identification (BCID) panel (bioMérieux, Marcy l’Etoile, France) is a multiplex PCR assay with 2 min of hands-on time and a 1 h turnaround time that allows for syndromic diagnosis of bloodstream infection (BSI). The BioFire FilmArray BCID2 panel encompasses 43 molecular targets associated with BSI, including 15 Gram-negative bacteria, 11 Gram-positive bacteria, seven yeast species, and 10 antimicrobial resistance genes. The BCID2 Panel demonstrated high diagnostic accuracy compared to conventional microbiological methods [58,59,60,61].

If blood cultures give no positive results, the use of molecular tests, such as a polymerase chain reaction, can help clinicians rapidly (<2 h) identify organisms. These tests may target a single organism (i.e., *S. aureus*) or large panels of pathogens [62].

Using these panels can significantly reduce the time of identification compared to BC, providing reliable support to confirm or exclude a bloodstream infection. Integrating these tests and BC could help overcome current diagnostic issues, even in neonates where clinical vulnerability requires a rapid and sensitive approach [63].

Since previous antibiotic treatment is another known confounder for BC performance, which can decrease the identification rate when administered before BC collection [64], molecular tests can increase the possibility of detecting pathogens [63].

This can help to narrow the spectrum of the antimicrobial treatment and avoid prolonging anti-Gram-positive drugs in the case of Gram-negative bacteria, or vice versa, limiting the development of antibiotic resistance.

## 6. Antimicrobial Time Out and Automatic Stop Order (es. 48 h)

To lower drug exposure and abuse [65,66,67,68], the US Center for Disease Control and Prevention’s “Get Smart for Healthcare” program proposed a 48 h automatic antibiotic stop order for specified antibiotic prescriptions, especially for antibiotics administered for surgical prophylaxis [69].

Tolia et al. observed a reduced median antibiotic exposure after introducing an antibiotic automatic stop order (ASO) in the first seven days of life. With this ASO, antibiotics were automatically discontinued after the prespecified “end dose” unless actively reordered by the clinician, which would require an entirely new order. There was no provider alert or notification that the antibiotic order was expiring. These changes were recommended for all antibiotics started on admission, and providers could modify these written orders based on their discretion. This implementation was not associated with an increase in adverse events; however, the use of this ASO was predicated on the observation that EOS is uncommon, and the effect of this early ASO on later antibiotic usage and other clinical outcomes remains to be prospectively confirmed [70].

Similarly, Astorga et al. demonstrated that an automatic 48 h antibiotic stop order was highly effective at decreasing antibiotic usage, and with fewer doses of certain antibiotics (such as vancomycin), but always concerning early onset sepsis [71].

Other authors confirmed these results in early onset sepsis, suggesting that implementing an ASO is achievable by designing specific order sets within existing electronic medical record systems. These measures should be simple to implement and apply in most NICU settings [72,73,74,75,76].

However, similar results in late-onset sepsis are still unavailable.

## 7. Shortest Effective Duration of Therapy

Prolonged and unneeded antibiotic use, particularly in low-income countries, promotes the emergence of MDR bacteria [77]. Furthermore, a long course of antibiotics changes the natural individual’s microbiome and may result in subsequent difficult-to-treat infections [78]. The shortest course of therapy is an option that should be explored; however, to date, few studies have examined the effectiveness of shorter antibiotic courses, and there is no consensus on the appropriate duration of antibiotic treatment for culture-proven uncomplicated neonatal sepsis [79,80].

Recently, a systematic review analyzed the current evidence about a short course of intravenous antibiotics in treating culture-proven uncomplicated neonatal sepsis, including 447 infants born with a gestational age greater than 32 weeks (223 patients in the intervention arms and 224 in the control arms). A short duration of antibiotics was defined as receiving antibiotics targeting the isolated microorganism for equal to or more than 7–10 days, whereas the standard course refers to more than 10–14 days. Five randomized controlled trials, all conducted in India, included 51.6% of EOS episodes and 48.4% of LOS episodes in the short-duration arm [80].

Only three studies reported all-cause mortality within 28 days after discharge or completion of antibiotic treatment, with an odds ratio of 0.32 (95% CI 0.01–8.24) for a short course compared to a standard duration. The evidence was very low due to a high risk of bias across almost all studies [80].

Afterward, Islam et al. conducted a new RCT in India, enrolling 115 patients in the intervention arm (ten-day antibiotic therapy) and 116 in the control group (14-day antibiotic therapy), again including both EOS and LOS episodes. There were no deaths in the two groups. They found no significant differences in treatment failure among the two groups, with a significantly lower duration of hospital stay in the study group [79]. However, they excluded the critically ill and very low birth weight neonates, and thus it is difficult to draw definitive conclusions about practicing a shorter duration of antibiotics.

Currently, a multicenter randomized controlled non-inferiority trial (Clinical Trials ID: NCT03280147) conducted in India is going to compare the efficacy of a 7-day versus a 14-day course of intravenous antibiotics in the treatment of uncomplicated neonatal bacterial sepsis among neonates weighing >1000 g at birth with culture-proven bacterial sepsis that is uncomplicated by meningitis, bone or joint infections, or deep-seated abscesses. The primary outcome measure will be a definite or probable relapse within 21 days after the stoppage of antibiotics [81]. To date, they enrolled 261 infants (https://clinicaltrials.gov/study/NCT03280147, accessed on 20 January 2024).

Multicenter studies, if possible, across different countries (including both high-income and low-income ones) and separately (including EOS and LOS episodes) would be crucial to confirm that a short course of antibiotics is a safe option in culture-proven sepsis.

## 8. The Transition from Intravenous to Oral Treatment

When clinically warranted, and as soon as possible, antimicrobial regimens should be changed from intravenous to oral delivery, as in adults.

Oral antibiotics given to neonates are absorbed and provide appropriate blood levels over time, as determined by the minimum inhibitory concentrations (MICs) of relevant infections. Efficacy studies are encouraging without any reinfection or treatment failure, although robust evidence is still lacking. Both families and healthcare systems may benefit from early oral antibiotic switch treatment in newborns [82]. Indeed, longer periods of intravenous antibiotic treatment could expose infants to further risks (such as administration errors and further healthcare-associated infections due to the selection of antibiotic-resistant strains) and increase the length of hospital stay and stress to infants and their families.

Recently, the multicenter, randomized, open-label, non-inferiority trial “Reduction of intravenous Antibiotics In Neonates” (RAIN study) yielded results about the efficacy and safety of switching from intravenous to oral antibiotics (amoxicillin-clavulanic acid) versus a full course of intravenous antibiotics in neonates with probable bacterial infection. In a cohort of 510 neonates who received prolonged antibiotic treatment, they showed that an intravenous-to-oral switch was not inferior to a full course of intravenous antibiotics. However, infants had early onset sepsis in 97% and 98% of cases in the oral amoxicillin-clavulanic acid group and in the intravenous antibiotics arm, respectively [83].

Therefore, the number of included infants with LOS does not allow us to draw any definitive conclusions about LOS management. Additional well-designed studies targeted at LOS cases are required.

## 9. The Role of Lumbar Puncture in the Evaluation for Neonatal Sepsis

Neonates are more susceptible to meningitis and could not exhibit typical symptoms like seizures or a protruding fontanelle. Therefore, in certain situations, a lumbar puncture (LP) should be considered while evaluating a baby for sepsis [84]. Although meningitis in these patients is more likely when bacteremia is present, around one-third of instances of meningitis happen when blood cultures are normal in preterm infants [85].

A crucial problem is that LP is often performed after antibiotic administration, hampering the ability to identify the pathogen in the cerebrospinal fluid (CSF) culture. However, also considering that polymerase chain reaction increases the probability of positive results of CSF analysis compared to microbiological culture alone, LP should be performed before the beginning of antibiotics if possible [86].

Lumbar puncture in the neonatal population is a safe procedure, whereas the implications of missing meningitis are potentially very serious [87]. If performed, LP has surely a role in determining the length of antibiotic therapy. In the case of culture-proven meningitis, the suggested duration is usually 14–21 days, with at least 21 days recommended for *E. coli* and other meningitis caused by Gram-negative bacilli [88]. Positive experiences regarding a shorter duration of antibiotic therapy (10 days) are starting to appear, but further studies on larger samples are needed [89]. In cases of negative microbiological CSF results and improved clinical conditions, antibiotic therapy could be safely stopped.

Furthermore, newborns may present a septic syndrome not caused by bacteria, as in cases of *Enterovirus*/*Parechovirus* encephalitis, and therefore, an early analysis of the CSF can save unrequired antibiotics, direct towards therapy with intravenous immunoglobulins, and lead to the correct identification of brain MRI abnormalities, which a simple cranial ultrasound would not identify [90]. However, it is also true that this type of infection is the prerogative of outpatients and is not generally contracted within the NICU.

## 10. Conclusions

Currently, inappropriate and excessive use of antibiotics is observed among humans and food-producing animals. Italy is one of the European Union countries with the highest consumption of antibiotics, as, in the human sector, the average consumption is 10% higher than the European average [91].

NICUs represent one of the care departments where antibiotic pressure is greatest. Each intensive care unit should have a group of professionals, doctors, and nurses dedicated, with a multidisciplinary approach, to implementing antibiotic stewardship, i.e., those activities aimed at surveillance, verification, and continuous staff training on the appropriate use of antibiotics [92]. Using targeted antibiotic therapies, narrowing the spectrum of therapy as soon as possible, re-evaluating the real need for antibiotic therapy every 36–48 h, prudently reducing the duration of antibiotic therapies as much as possible, and using infection biomarkers appropriately can also help to achieve an antibiotic stewardship in neonatal departments.

When to start: clinical symptoms remain the starting point of the diagnostic process; biomarkers support doctors in the decision to promptly start antibiotic therapy in neonates when sepsis is suspected [93].

When to stop: After 36–48 h, if the blood cultures are negative and the baby is clinically stable. Nevertheless, doctors need protocols for biomarkers-guided antibiotic stewardship. To date, PCT is the marker with the best evidence of performance in this sense, as extrapolated from research on early onset cases. P-SEP also seems to be a promising discriminant of sepsis in neonates, but further studies are needed about the three biomarkers (CRP, PCT, and P-SEP) distinguishing EOS and LOS cases (often considered together in some studies).

Finally, most of all, all of us must be antimicrobial stewards.

## Figures and Tables

**Table 1:** Neonatal Sequential Organ Failure Assessment (nSOFA): the score ranges from 0 (best) to 15 (worst), as described by Wynn and Polin [12]. *FiO_2_: fraction of inspired oxygen; NA: not applicable; SpO_2_: oxygen saturation measured by pulse oximetry*.

**nSOFA**	**Respiratory score**	**0**	**2**	**4**	**6**	**8**
	criteria	Not intubated or intubated, SpO_2_/FiO_2_ ≥ 300	Intubated, SpO_2_/FiO_2_ <300	Intubated, SpO_2_/FiO_2_ <200	Intubated, SpO_2_/FiO_2_ <150	Intubated, SpO_2_/FiO_2_ <100
	**Cardiovascular score**	**0**	**1**	**2**	**3**	**4**
	criteria	No inotropes, no systemic steroids	No inotropes, systemic steroid treatment	One inotrope, no systemic steroids	At least 2 inotropes, or one inotrope and systemic steroids	At least two inotropes and systemic steroids
	**Hematologic score**	**0**	**1**	**2**	**3**	**NA**
	criteria	Platelet count ≥ 150 × 10^9^ L	Platelet count 100–149 × 10^9^ L	Platelet count <100 × 10^9^ L	Platelet count <50 × 10^9^ L	

**Table 2:** NeoSep Severity Score, as described by Russell et al. [16].

Factors		Severity Score Points
**Birthweight**	<1 kg	**2**
	1.0–2.9 kg	**1**
	>3 kg	**0**
**In-hospital time**	≤10 days: 1	**1**
	>10 days	**0**
**Gestational age**	<37 weeks	**1**
**Congenital anomalies**	Yes	**1**
	No	**0**
**Maximum respiratory support**	Oxygen supplementation	**2**
	Non-invasive ventilation	**3**
	Invasive ventilation	**3**
**Body temperature**	<35.5 °C	**1**
	35.6–37.9 °C	**0**
	≥38–<39 °C	**1**
	≥39 °C	**2**
**Abdominal distension**	Yes	**1**
	No	**0**
**Lethargy, no or reduced movement**	No	**0**
	Lethargy only	**1**
	No/reduced movements	**2**
**Feeding difficulties**	Yes	**1**
	No	**0**
**Evidence of shock**	Yes	**1**
	No	**0**
**Maximum number of score possible**		**16**

**Table 3:** NeoSep Recovery Score, as described by Russell et al. [16].

Factors		Severity Score Points
**Maximum respiratory support**	Oxygen supplementation	**2**
	Non-invasive ventilation	**3**
	Invasive ventilation	**3**
**Body temperature**	<35.5 °C	**1**
	35.6–37.9 °C	**0**
	≥38–<39 °C	**1**
	≥39 °C	**2**
**Abdominal distension**	Yes	**1**
	No	**0**
**Lethargy, no or reduced movement**	No	**0**
	Lethargy only	**1**
	No/reduced movements	**2**
**Feeding difficulties**	Yes	**1**
	No	**0**
**Evidence of shock**	Yes	**1**
	No	**0**
**Cyanosis**	Yes	**1**
	No	**0**
**Maximum number of score possible**		**11**

## Data Availability

Articles considered for the review of literature are already available on PubMed.

## References

[1] Stocker M., Klingenberg C., Navér L., Nordberg V., Berardi A., el Helou S., Fusch G., Bliss J.M., Lehnick D., Dimopoulou V. (2023). Less Is More: Antibiotics at the Beginning of Life. Nat. Commun..

[2] Prusakov P., Goff D.A., Wozniak P.S., Cassim A., Scipion C.E.A., Urzúa S., Ronchi A., Zeng L., Ladipo-Ajayi O., Aviles-Otero N. (2021). A Global Point Prevalence Survey of Antimicrobial Use in Neonatal Intensive Care Units: The No-More-Antibiotics and Resistance (NO-MAS-R) Study. EClinicalMedicine.

[3] Russell N., Barday M., Okomo U., Dramowski A., Sharland M., Bekker A. (2023). Early-versus Late-Onset Sepsis in Neonates—Time to Shift the Paradigm?. Clin. Microbiol. Infect..

[4] Achten N.B., Klingenberg C., Benitz W.E., Stocker M., Schlapbach L.J., Giannoni E., Bokelaar R., Driessen G.J.A., Brodin P., Uthaya S. (2019). Association of Use of the Neonatal Early-Onset Sepsis Calculator with Reduction in Antibiotic Therapy and Safety A Systematic Review and Meta-Analysis. JAMA Pediatr..

[5] Hayes R., Hartnett J., Semova G., Murray C., Murphy K., Carroll L., Plapp H., Hession L., O’Toole J., McCollum D. (2023). Neonatal Sepsis Definitions from Randomised Clinical Trials. Pediatr. Res..

[6] Wynn J.L. (2016). Defining Neonatal Sepsis. Curr. Opin. Pediatr..

[7] Flannery D.D., Ross R.K., Mukhopadhyay S., Tribble A.C., Puopolo K.M., Gerber J.S. (2018). Temporal Trends and Center Variation in Early Antibiotic Use Among Premature Infants. JAMA Netw. Open.

[8] Katz S., Banerjee R., Schwenk H. (2021). Antibiotic Stewardship for the Neonatologist and Perinatologist. Clin. Perinatol..

[9] Singer M., Deutschman C.S., Seymour C., Shankar-Hari M., Annane D., Bauer M., Bellomo R., Bernard G.R., Chiche J.D., Coopersmith C.M. (2016). The Third International Consensus Definitions for Sepsis and Septic Shock (Sepsis-3). JAMA.

[10] Hofer N., Zacharias E., Müller W., Resch B. (2012). Performance of the Definitions of the Systemic Inflammatory Response Syndrome and Sepsis in Neonates. J. Perinat. Med..

[11] Bromiker R., Elron E., Klinger G. (2020). Do Neonatal Infections Require a Positive Blood Culture?. Am. J. Perinatol..

[12] Wynn J.L., Polin R.A. (2020). A Neonatal Sequential Organ Failure Assessment Score Predicts Mortality to Late-Onset Sepsis in Preterm Very Low Birth Weight Infants. Pediatr. Res..

[13] Fleiss N., Coggins S.A., Lewis A.N., Zeigler A., Cooksey K.E., Walker L.A., Husain A.N., De Jong B.S., Wallman-Stokes A., Alrifai M.W. (2021). Evaluation of the Neonatal Sequential Organ Failure Assessment and Mortality Risk in Preterm Infants with Late-Onset Infection. JAMA Netw. Open.

[14] Poggi C., Ciarcià M., Miselli F., Dani C. (2023). Prognostic Accuracy of Neonatal SOFA Score versus SIRS Criteria in Preterm Infants with Late-Onset Sepsis. Eur. J. Pediatr..

[15] Sokou R., Ioakeimidis G., Piovani D., Parastatidou S., Konstantinidi A., Tsantes A.G., Lampridou M., Houhoula D., Iacovidou N., Kokoris S. (2022). Development and Validation of a Sepsis Diagnostic Scoring Model for Neonates with Suspected Sepsis. Front. Pediatr..

[16] Russell N.J., Stohr W., Plakka N., Cook A., Berkley J.A., Adhisivam B., Agarwal R., Ahmed N.U., Balasegaram M., Ballot D. (2023). Patterns of Antibiotic Use, Pathogens, and Prediction of Mortality in Hospitalized Neonates and Young Infants with Sepsis: A Global Neonatal Sepsis Observational Cohort Study (NeoOBS). PLoS Med..

[17] Schmatz M., Srinivasan L., Grundmeier R.W., Elci O.U., Weiss S.L., Masino A.J., Tremoglie M., Ostapenko S., Harris M.C. (2020). Surviving Sepsis in a Referral Neonatal Intensive Care Unit: Association between Time to Antibiotic Administration and In-Hospital Outcomes. J. Pediatr..

[18] Dong Y., Speer C.P. (2015). Late-Onset Neonatal Sepsis: Recent Developments. Arch. Dis. Child Fetal Neonatal Ed..

[19] Cantey J.B., Lee J.H. (2021). Biomarkers for the Diagnosis of Neonatal Sepsis. Clin. Perinatol..

[20] Pons S., Trouillet-Assant S., Subtil F., Abbas-Chorfa F., Cornaton E., Berthiot A., Galletti S., Plat A., Rapin S., Trapes L. (2023). Performance of 11 Host Biomarkers Alone or in Combination in the Diagnosis of Late-Onset Sepsis in Hospitalized Neonates: The Prospective EMERAUDE Study. Biomedicines.

[21] Maddaloni C., De Rose D.U., Santisi A., Martini L., Caoci S., Bersani I., Ronchetti M.P., Auriti C. (2021). The Emerging Role of Presepsin (P-Sep) in the Diagnosis of Sepsis in the Critically Ill Infant: A Literature Review. Int. J. Mol. Sci..

[22] Maddaloni C., De Rose D.U., Perulli M., Martini L., Bersani I., Campi F., Savarese I., Dotta A., Paola M., Cinzia R. (2023). Perinatal Asphyxia Does Not Influence Presepsin Levels in Neonates: A Prospective Study. Acta Paediatr..

[23] Stocker M., Giannoni E. (2023). Game Changer or Gimmick: Inflammatory Markers to Guide Antibiotic Treatment Decisions in Neonatal Early-Onset Sepsis. Clin. Microbiol. Infect..

[24] Brown J.V.E., Meader N., Wright K., Cleminson J., McGuire W. (2020). Assessment of C-Reactive Protein Diagnostic Test Accuracy for Late-Onset Infection in Newborn Infants: A Systematic Review and Meta-Analysis. JAMA Pediatr..

[25] Glaser M.A., Hughes L.M., Jnah A., Newberry D., Harris-Haman P.A. (2021). Neonatal Sepsis: A Review of Pathophysiology and Current Management Strategies. Adv. Neonatal Care.

[26] Stocker M., Van Herk W., Helou S., Dutta S., Fontana M.S., Schuerman F.A.B.A., Groot R.K.V.D.T. (2017). Procalcitonin-Guided Decision Making for Duration of Antibiotic Therapy in Neonates with Suspected Early-Onset Sepsis: A Multicentre, Randomised Controlled Trial (NeoPIns). Lancet.

[27] NICE Guidelines Neonatal Infection: Antibiotics for Prevention and Treatment. Neonatal Infection: Antibiotics for Prevention and Treatment 2021. https://www.nice.org.uk/guidance/ng195.

[28] Meem M., Modak J.K., Mortuza R., Morshed M., Islam M.S., Saha S.K. (2011). Biomarkers for Diagnosis of Neonatal Infections: A Systematic Analysis of Their Potential as a Point-of-Care Diagnostics. J. Glob. Health.

[29] Ehl S., Gering B., Bartmann P., Högel J., Pohlandt F. (1997). C-Reactive Protein Is a Useful Marker for Guiding Duration of Antibiotic Therapy in Suspected Neonatal Bacterial Infection. Pediatrics.

[30] Benitz W.E., Yan M.Y., Madan A., Ramachandra P. (1998). Serial Serum C-Reactive Protein Levels in the Diagnosis of Neonatal Infection. Pediatrics.

[31] University of Pennsylvania Study “Using Biomarkers to Optimize Antibiotic Strategies in Sepsis” (ID: NCT02207114). NCT02207114.

[32] Aloisio E., Dolci A., Panteghini M. (2019). Procalcitonin: Between Evidence and Critical Issues. Clin. Chim. Acta.

[33] Assistance Publique—Hôpitaux de Paris Study “Procalcitonin and Duration of AntiBiotherapy in Late Onset Sepsis of Neonate (PROABIS)” (ID: NCT03730636). NCT03730636.

[34] Eichberger J., Resch B. (2022). Reliability of Interleukin-6 Alone and in Combination for Diagnosis of Early Onset Neonatal Sepsis: Systematic Review. Front. Pediatr..

[35] Küng E., Unterasinger L., Waldhör T., Berger A., Wisgrill L. (2023). Cut-off Values of Serum Interleukin-6 for Culture-Confirmed Sepsis in Neonates. Pediatr. Res..

[36] Ng P.C., Cheng S.H., Chui K.M., Fok T.F., Wong M.Y., Wong W., Wong R.P.O., Cheung K.L. (1997). Diagnosis of Late Onset Neonatal Sepsis with Cytokines, Adhesion Molecule, and C-Reactive Protein in Preterm Very Low Birthweight Infants. Arch. Dis. Child Fetal Neonatal Ed..

[37] Chauhan N., Tiwari S., Jain U. (2017). Potential Biomarkers for Effective Screening of Neonatal Sepsis Infections: An Overview. Microb. Pathog..

[38] Zhou M., Cheng S., Yu J., Lu Q. (2015). Interleukin-8 for Diagnosis of Neonatal Sepsis: A Meta-Analysis. PLoS ONE.

[39] Franz A.R., Steinbach G., Kron M., Pohlandt F. (2001). Interleukin-8: A Valuable Tool to Restrict Antibiotic Therapy in Newborn Infants. Acta Paediatr..

[40] Carpio R., Zapata J., Spanuth E., Hess G. (2015). Utility of Presepsin (SCD14-ST) as a Diagnostic and Prognostic Marker of Sepsis in the Emergency Department. Clin. Chim. Acta.

[41] Capossela L., Margiotta G., Ferretti S., Curatola A., Bertolaso C., Pansini V., Di Sarno L., Gatto A. (2023). Presepsin as a Diagnostic Marker of Sepsis in Children and Adolescents: A Short Critical Update. Acta Biomed..

[42] Bellos I., Fitrou G., Pergialiotis V., Thomakos N., Perrea D.N., Daskalakis G. (2018). The Diagnostic Accuracy of Presepsin in Neonatal Sepsis: A Meta-Analysis. Eur. J. Pediatr..

[43] Pietrasanta C., Ronchi A., Vener C., Poggi C., Ballerini C., Testa L., Colombo R.M., Spada E., Dani C., Mosca F. (2021). Presepsin (Soluble Cd14 Subtype) as an Early Marker of Neonatal Sepsis and Septic Shock: A Prospective Diagnostic Trial. Antibiotics.

[44] Poggi C., Bianconi T., Gozzini E., Generoso M., Dani C. (2015). Presepsin for the Detection of Late-Onset Sepsis in Preterm Newborns. Pediatrics.

[45] Sabry J.H., Elfeky O.A., Elsadek A.E., Eldaly A.A. (2016). Presepsin as an Early Reliable Diagnostic and Prognostic Marker of Neonatal Sepsis. Int. J. Adv. Res..

[46] Miyosawa Y., Akazawa Y., Kamiya M., Nakamura C., Takeuchi Y., Kusakari M., Nakamura T. (2018). Presepsin as a Predictor of Positive Blood Culture in Suspected Neonatal Sepsis. Pediatr. Int..

[47] Fabre V., Carroll K.C., Cosgrove S.E. (2022). Blood Culture Utilization in the Hospital Setting: A Call for Diagnostic Stewardship. J. Clin. Microbiol..

[48] Huber S., Hetzer B., Crazzolara R., Orth-Höller D. (2020). The Correct Blood Volume for Paediatric Blood Cultures: A Conundrum?. Clin. Microbiol. Infect..

[49] Hajjar N., Ting J.Y., Shah P.S., Lee K.-S., Dunn M.S., Srigley J.A., Khurshid F. (2023). Blood Culture Collection Practices in NICU; A National Survey. Paediatr. Child Health.

[50] Coggins S.A., Harris M.C., Srinivasan L. (2022). Dual-Site Blood Culture Yield and Time to Positivity in Neonatal Late-Onset Sepsis. Arch. Dis. Child Fetal Neonatal Ed..

[51] Kaiser J.R., Cassat J.E., Lewno M.J. (2002). Should Antibiotics Be Discontinued at 48 Hours for Negative Late-Onset Sepsis Evaluations in the Neonatal Intensive Care Unit?. J. Perinatol..

[52] Mukhopadhyay S., Briker S.M., Flannery D.D., Dhudasia M.B., Coggins S.A., Woodford E., Walsh E.M., Li S., Puopolo K.M., Kuzniewicz M.W. (2022). Time to Positivity of Blood Cultures in Neonatal Late-Onset Bacteraemia. Arch. Dis. Child Fetal Neonatal Ed..

[53] Guerti K., Devos H., Ieven M.M., Mahieu L.M. (2011). Time to Positivity of Neonatal Blood Cultures: Fast and Furious?. J. Med. Microbiol..

[54] Abdelhamid S. (2017). Time to Positivity and Antibiotic Sensitivity of Neonatal Blood Cultures. J. Glob. Infect. Dis..

[55] Dierig A., Berger C., Agyeman P.K.A., Bernhard-Stirnemann S., Giannoni E., Stocker M., Posfay-Barbe K.M., Niederer-Loher A., Kahlert C.R., Donas A. (2018). Time-to-Positivity of Blood Cultures in Children with Sepsis. Front. Pediatr..

[56] Mokrani D., Chommeloux J., Pineton de Chambrun M., Hékimian G., Luyt C.E. (2023). Antibiotic Stewardship in the ICU: Time to Shift into Overdrive. Ann. Intensive Care.

[57] Oeser C., Pond M., Butcher P., Bedford Russell A., Henneke P., Laing K., Planche T., Heath P.T., Harris K. (2020). PCR for the detection of pathogens in neonatal early onset sepsis. PLoS ONE.

[58] Peri A.M., Ling W., Furuya-Kanamori L., Harris P.N.A., Paterson D.L. (2022). Performance of BioFire Blood Culture Identification 2 Panel (BCID2) for the Detection of Bloodstream Pathogens and Their Associated Resistance Markers: A Systematic Review and Meta-Analysis of Diagnostic Test Accuracy Studies. BMC Infect. Dis..

[59] Graff K.E., Palmer C., Anarestani T., Velasquez D., Hamilton S., Pretty K., Parker S., Dominguez S.R. (2021). Clinical Impact of the Expanded BioFire Blood Culture Identification 2 Panel in a U.S. Children’s Hospital. Microbiol. Spectr..

[60] Berinson B., Both A., Berneking L., Christner M., Lütgehetmann M., Aepfelbacher M., Rohde H. (2021). Usefulness of Biofire Filmarray Bcid2 for Blood Culture Processing in Clinical Practice. J. Clin. Microbiol..

[61] Messacar K., Hurst A.L., Child J., Campbell K., Palmer C., Hamilton S., Dowell E., Robinson C.C., Parker S.K., Dominguez S.R. (2017). Clinical Impact and Provider Acceptability of Real-Time Antimicrobial Stewardship Decision Support for Rapid Diagnostics in Children with Positive Blood Culture Results. J. Pediatr. Infect. Dis. Soc..

[62] Gonzalez M.D., Chao T., Pettengill M.A. (2020). Modern Blood Culture: Management Decisions and Method Options. Clin. Lab Med..

[63] Lucignano B., Cento V., Agosta M., Ambrogi F., Albitar-Nehme S., Mancinelli L., Mattana G., Onori M., Galaverna F., Di Chiara L. (2022). Effective Rapid Diagnosis of Bacterial and Fungal Bloodstream Infections by T2 Magnetic Resonance Technology in the Pediatric Population. J. Clin. Microbiol..

[64] Scheer C.S., Fuchs C., Gründling M., Vollmer M., Bast J., Bohnert J.A., Zimmermann K., Hahnenkamp K., Rehberg S., Kuhn S.O. (2019). Impact of Antibiotic Administration on Blood Culture Positivity at the Beginning of Sepsis: A Prospective Clinical Cohort Study. Clin. Microbiol. Infect..

[65] Isaacs D. (2006). Unnatural Selection: Reducing Antibiotic Resistance in Neonatal Units. Arch. Dis. Child Fetal Neonatal Ed..

[66] Cantey J.B., Sánchez P.J. (2011). Prolonged Antibiotic Therapy for “Culture-Negative” Sepsis in Preterm Infants: It’s Time to Stop!. J. Pediatr..

[67] Falciglia G., Hageman J.R., Schreiber M., Alexander K. (2012). Antibiotic Therapy and Early Onset Sepsis. Neoreviews.

[68] Ho T., Dukhovny D., Zupancic J.A.F., Goldmann D.A., Horbar J.D., Pursley D.M. (2015). Choosing Wisely in Newborn Medicine: Five Opportunities to Increase Value. Pediatrics.

[69] Centers for Disease Control and Prevention (CDC) Core Elements of Hospital Antibiotic Stewardship Programs. https://www.cdc.gov/antibiotic-use/core-elements/hospital.html.

[70] Tolia V.N., Desai S., Qin H., Rayburn P.D., Poon G., Murthy K., Ellsbury D.L., Chiruvolu A. (2017). Implementation of an Automatic Stop Order and Initial Antibiotic Exposure in Very Low Birth Weight Infants. Am. J. Perinatol..

[71] Astorga M.C., Piscitello K.J., Menda N., Ebert A.M., Ebert S.C., Porte M.A., Kling P.J. (2019). Antibiotic Stewardship in the Neonatal Intensive Care Unit: Effects of an Automatic 48-Hour Antibiotic Stop Order on Antibiotic Use. J. Pediatr. Infect. Dis. Soc..

[72] Cantey J.B., Wozniak P.S., Pruszynski J.E., Sánchez P.J. (2016). Reducing Unnecessary Antibiotic Use in the Neonatal Intensive Care Unit (SCOUT): A Prospective Interrupted Time-Series Study. Lancet Infect. Dis..

[73] Lu C., Liu Q., Yuan H., Wang L. (2019). Implementation of the Smart Use of Antibiotics Program to Reduce Unnecessary Antibiotic Use in a Neonatal ICU: A Prospective Interrupted Time-Series Study in a Developing Country. Crit. Care Med..

[74] Wang B., Li G., Jin F., Weng J., Peng Y., Dong S., Liu J., Luo J., Wu H., Shen Y. (2020). Effect of Weekly Antibiotic Round on Antibiotic Use in the Neonatal Intensive Care Unit as Antibiotic Stewardship Strategy. Front. Pediatr..

[75] Singh N., Gray J.E. (2021). Antibiotic Stewardship in NICU: De-Implementing Routine CRP to Reduce Antibiotic Usage in Neonates at Risk for Early-Onset Sepsis. J. Perinatol..

[76] Muller M.R., Mahadeo A.M., Mayne J.P., Mennella J.M., Mun P.A., Tucker R., Bliss J.M. (2022). Decreased Antibiotic Exposure for Suspected Early-Onset Sepsis in the Neonatal Intensive Care Unit Through Implementation of an Antimicrobial Time-Out. J. Pediatr. Pharmacol.Ther..

[77] Chaurasia S., Sivanandan S., Agarwal R., Ellis S., Sharland M., Sankar M.J. (2019). Neonatal Sepsis in South Asia: Huge Burden and Spiralling Antimicrobial Resistance. BMJ.

[78] Duan H., Yu L., Tian F., Zhai Q., Fan L., Chen W. (2022). Antibiotic-Induced Gut Dysbiosis and Barrier Disruption and the Potential Protective Strategies. Crit. Rev. Food Sci. Nutr..

[79] Islam K., Khatun N., Das K., Paul S., Ghosh T., Nayek K. (2023). Ten- vs. 14-Day Antibiotic Therapy for Culture-Positive Neonatal Sepsis. J. Trop. Pediatr..

[80] Aljarbou A., Cuello C., Leslie A.T.F.S. (2023). Short Course of Intravenous Antibiotics in the Treatment of Uncomplicated Proven Neonatal Bacterial Sepsis: A Systematic Review. Acta Paediatr..

[81] Dutta S., Nangia S., Jajoo M., Gathwala G., Nesargi S., Sundaram M., Kumar P., Saili A., Kumar D., Dalal P. (2021). Comparison of Efficacy of a 7-Day versus a 14-Day Course of Intravenous Antibiotics in the Treatment of Uncomplicated Neonatal Bacterial Sepsis: Study Protocol of a Randomized Controlled Non-Inferiority Trial. Trials.

[82] Keij F.M., Kornelisse R.F., Hartwig N.G., Reiss I.K.M., Allegaert K., Tramper-Stranders G.A. (2019). Oral Antibiotics for Neonatal Infections: A Systematic Review and Meta-Analysis. J. Antimicrob. Chemother..

[83] Keij F.M., Kornelisse R.F., Hartwig N.G., van der Sluijs-Bens J., van Beek R.H.T., van Driel A., van Rooij L.G.M., van Dalen-Vink I., Driessen G.J.A., Kenter S. (2022). Efficacy and Safety of Switching from Intravenous to Oral Antibiotics (Amoxicillin–Clavulanic Acid) versus a Full Course of Intravenous Antibiotics in Neonates with Probable Bacterial Infection (RAIN): A Multicentre, Randomised, Open-Label, Non-Inferiorit. Lancet Child Adolesc. Health.

[84] Aleem S., Greenberg R.G. (2019). When to include a lumbar puncture in the evaluation for neonatal sepsis. Neoreviews.

[85] Smith P.B., Garges H.P., Cotton C.M., Walsh T.J., Clark R.H., Benjamin D.K. (2008). Meningitis in preterm neonates: Importance of cerebrospinal fluid parameters. Am. J. Perinatol..

[86] Bedetti L., Miselli F., Minotti C., Latorre G., Loprieno S., Foglianese A., Laforgia N., Perrone B., Ciccia M., Capretti M.G. (2023). Lumbar Puncture and Meningitis in Infants with Proven Early- or Late-Onset Sepsis: An Italian Prospective Multicenter Observational Study. Microorganisms.

[87] Malbon K., Mohan R., Nicholl R. (2006). Should a neonate with possible late onset infection always have a lumbar puncture?. Arch. Dis. Child..

[88] Ting J.Y., Autmizguine J., Dunn M.S., Choudhury J., Blackburn J., Gupta-Bhatnagar S., Assen K., Emberley J., Khan S., Leung J. (2022). Practice Summary of Antimicrobial Therapy for Commonly Encountered Conditions in the Neonatal Intensive Care Unit: A Canadian Perspective. Front Pediatr..

[89] Mathur N.B., Kharod P., Kumar S. (2015). Evaluation of duration of Antibiotic Therapy in Neonatal Bacterial Meningitis: A randomized controlled trial. J. Trop. Pediatr..

[90] Bucci S., Coltella L., Martini L., Santisi A., De Rose D.U., Piccioni L., Campi F., Ronchetti M.P., Longo D., Lucignani G. (2022). Clinical and Neurodevelopmental Characteristics of Enterovirus and Parechovirus Meningitis in Neonates. Front. Pediatr..

[91] (2021). AIFA—Italian Agency for Drugs: The Medicines Utilisation Monitoring Centre Antibiotics. In: National Report on Drugs Use in Italy. https://www.aifa.gov.it/-/l-uso-dei-farmaci-in-italia-rapporto-osmed-2021.

[92] Fleiss N., Hooven T.A., Polin R.A. (2021). Can We Back off Using Antibiotics in the NICU?. Semin. Fetal Neonatal Med..

[93] Cohen R., Romain O., Tauzin M., Gras-Leguen C., Raymond J., Butin M. (2023). Neonatal Bacterial Infections: Diagnosis, Bacterial Epidemiology and Antibiotic Treatment. Infect. Dis. Now.

